# Analysis of phosphomotifs coupled to phosphoproteome and interactome unveils potential human kinase substrate proteins in SARS-CoV-2

**DOI:** 10.3389/fcimb.2025.1554760

**Published:** 2025-07-09

**Authors:** Vineetha Shaji, Ahmad Rafi, Mukhtar Ahmed, Athira Perunelly Gopalakrishnan, Sowmya Soman, Amjesh Revikumar, Ganesh Prasad, Abhithaj Jayanandan, Rajesh Raju

**Affiliations:** ^1^ Centre for Integrative Omics Data Science, Yenepoya (Deemed to be University), Mangalore, India; ^2^ Centre for Systems Biology and Molecular Medicine, Yenepoya Research Centre, Yenepoya (Deemed to Be University), Mangalore, India; ^3^ Department of Zoology, College of Science, King Saud University, Riyadh, Saudi Arabia; ^4^ Department of Biochemistry, Yenepoya Medical College, Yenepoya (Deemed to be University), Mangalore, India

**Keywords:** phosphorylation, SARS-CoV-2, human kinase substrate motifs, human-viral protein-protein interactome, viral phosphosites

## Abstract

**Introduction:**

Viruses exploit host kinases to phosphorylate their proteins, enabling viral replication and interference with host-cell functions. Understanding phosphorylation in SARS-CoV-2 proteins necessitates identifying viral phosphoproteins, their phosphosites, and the host kinase–viral protein interactions critical for evading host antiviral responses.

**Methods:**

Employing the protein kinase substrate sequence-preference motifs derived by Poll B G. *et. al*., 2024, we performed kinase-substrate phosphomotif pattern analysis on the SARS-CoV-2 proteome. We identified major host kinases by analyzing SARS-CoV-2 perturbed phosphoproteomes from various studies and cell systems. These kinases were subjected to interactome analysis and literature-based validation for the impact of kinase inhibitors on infection. Further, conservation of viral phosphosites across SARS CoV-2 variants were also assessed.

**Results:**

The human kinome–substrate phosphomotif analysis predicted 49 kinases capable of phosphorylating 639 phosphosites across 33 SARS-CoV-2 proteins. From these, 24 kinases were also perturbed in SARS-CoV-2-infected phosphoproteomes. Literature review identified seven kinases, including MAP2K1, whose inhibition may reduce viral replication. MAP2K1 was found to target key viral phosphosites, including N protein (S206, T198) and ORF9b (S50), conserved across SARS-CoV-2 variants. Docking analysis showed MAP2K1 forms stronger, closer interactions with N protein compared to SRPK1, highlighting MAP2K1 as a potential host kinase for therapeutic targeting in SARS-CoV-2 infection.

**Discussion and Conclusions:**

This study presents a framework for predicting human kinases of specific SARS-CoV-2 protein phosphosites by integrating kinase specificity, virus–host interactions, and post-translational modifications. MAP2K1 was identified as a key host kinase, showing stronger interactions than SRPK1, and is proposed as an antiviral drug target for repurposing in SARS-CoV-2 infections.

## 1 Introduction

The emergence of the novel coronavirus Severe Acute Respiratory Syndrome Coronavirus 2 (SARS-CoV-2; 2019-nCoV) triggering the outbreak of coronavirus disease 2019 (COVID-19) has wreaked havoc on public health, a global pandemic that has posed significant challenges to human health (Zhu et al., 2024) (Pham et al., 2023). Among the several human coronaviruses which cause illness, only the SARS-CoV, MERS-CoV, and the novel SARS-CoV-2 are known to cause severe respiratory syndromes. The SARS-CoV-2 is considered particularly formidable due to its high transmissibility, even though its case fatality rate (~5%) is lower than that of SARS-CoV (~10%) and MERS-CoV (~37%) (Huang et al., 2020). Its genome contains fourteen open reading frames (ORFs), which are divided into two regions, ORF1a and ORF1ab, located in the first two-thirds of the viral genome (Arya et al., 2021). The ORF1a and ORF1ab polyproteins are cleaved by two viral proteases, papain-like protease (PLpro) and main protease (Mpro), leading to the production of sixteen nonstructural proteins (Nsps 1–16), which are essential for replication and transcription. Structural and accessory proteins, on the other hand, are translated from distinct subgenomic RNAs, with the exception of ORF9, which encodes the nucleocapsid (N) protein from the same subgenomic RNA (Zumla et al., 2016) (Yan et al., 2022). Subgenomic RNAs, synthesized by the viral RNA-dependent RNA polymerase, are translated using the host’s translation machinery into four structural proteins spike (S), membrane (M), envelope (E), and nucleocapsid (N) along with several accessory proteins including ORF3a, ORF3b, ORF6, ORF7a, ORF7b, ORF8, ORF9b, ORF9c, ORF10, ORF3d, and ORF3c (Harrison et al., 2020; Kakavandi et al., 2023; Michel et al., 2020; Stewart et al., 2023; Wu et al., 2020). Approximately one-third of the SARS-CoV-2 proteins have been identified or proposed to be phosphorylated at multiple sites in human cells, accompanied by significant modulation of phosphorylation in host cellular proteins, including various kinases (Stukalov et al., 2021; Hekman et al., 2021; Xiang et al., 2021; Klann et al., 2020; Davidson et al., 2020). SARS-CoV-2 infection disrupts host signaling pathways through protein–protein interactions between viral and human proteins (Gordon et al., 2020; Tutuncuoglu et al., 2020). Upon entering host cells, the virus hijacks cellular proteins to promote replication and evade immune responses, leading to significant dysregulation of cellular signaling (Durmus Tekir and Ulgen, 2013). However, the molecular and mechanistic links between protein interactions, phosphorylation responses, and changes in human kinase activities remains underexplored. The emergence of SARS-CoV-2 variants of concern (VOCs) such as Alpha, Beta, Gamma, Delta, and Omicron occurred independently, and each rapidly becoming dominant either regionally or globally, continues to outcompete previous variants (Carabelli et al., 2023). Arising independently, these variants exhibited mutations that enhanced transmissibility, altered viral properties, and in some cases, enabled immune evasion, allowing them to outcompete previous variants and spread rapidly. Understanding the unique characteristics of these VOCs is essential for uncovering key aspects of viral evolution, immune escape mechanisms, and the effectiveness of public health strategies, such as vaccination (Carabelli et al., 2023).

Phosphorylation, a key reversible post-translational modification, is critically involved in regulating a vast array of cellular processes in eukaryotic cells (Jakubiec and Jupin, 2007). It influences both host and viral proteins (Jacob et al., 2011). In humans and other mammals, it is mediated by over 520 protein kinases and mostly occurs on serine (S), threonine (T), or tyrosine (Y) residues within target proteins (Poll et al., 2024). Altered phosphorylation patterns indicate changes in kinase activities that are co-opted during infection (Bouhaddou et al., 2020). Several human kinases are reported to be perturbed upon SARS-CoV-2 infection, leading to extensive phosphorylation both in host cells and within the virus itself. Intriguingly, around 70 phosphorylation sites have been identified in SARS-CoV-2 viral proteins from different studies (Chatterjee and Thakur, 2022). Davidson et al., 2020, identified 44 phosphosites and over 500 viral peptides in SARS-CoV-2 infected cells, including those unique to a deleted spike protein variant (Davidson et al., 2020). Klann et al., 2020, identified 33 modification sites on six SARS-CoV-2 proteins, though their regulatory roles remain unclear. The study mapped host cell signaling networks during infection, highlighting pathways activated by SARS-CoV-2 (Klann et al., 2020). Hekman et al., 2020, identified eight SARS-CoV-2 viral proteins, including 2 phosphoproteins, with host-induced phosphorylation on viral proteins M and N. Nine phosphosites were detected on N, spanning the linker region between the RBD and dimerization domains, as well as in the C-terminal cytoplasmic domain (Hekman et al., 2020). Stukalov et al., 2021, reported 23 phosphorylation sites across five SARS-CoV-2 phosphoproteins (Stukalov et al., 2021). Identification of phosphorylation sites in viral proteins mediated by host kinases imply their potential as major therapeutic targets and significantly, identification of repurposable kinase inhibitors to combat the virus (Chatterjee and Thakur, 2022). With over 500 kinases known in humans and as phosphorylation is one of the major PTMs impacted by viruses, identification of potential phosphosites in viral proteins would open up a gateway for detection of human kinase-viral protein substrate interactions. For the majority of such phosphorylation events, responsible serine/threonine/tyrosine (Ser/Thr/Tyr) kinases encoded in the human genome is still unknown (Hornbeck et al., 2019).

Given the importance of kinase-substrate interactions and as significant changes in the phosphorylation of both host and viral proteins were observed following SARS-CoV-2 infection, identifying phosphorylation sites in SARS-CoV-2 proteins is crucial for understanding viral pathogenesis. A number of phosphorylation sites and kinase prediction tools are available to researchers (Gopalakrishnan et al., 2025) (Chen et al., 2020; Blom et al., 1999). However, among the large number of such human kinase-viral protein substrate pairs, filtering relevant pairs for targeted validation further is a challenge. Unbiased phosphoproteomics studies have generated extensive data useful for identifying protein kinase targets and their preferred substrate sequences. Data from Sugiyama et al., 2019., Poll et al. (2024) predicted sequence preference motifs for 384 protein kinases, offering valuable insights into their specific motif preferences (Hornbeck et al., 2019; Sugiyama et al., 2019; Poll et al., 2024). These human kinome substrate phosphomotifs can be engaged to predict potential interactions between human kinases and viral proteins.

The present study addresses this challenge by entailing computational tools and experimentally derived datasets to first systematically narrow down phosphorylation sites in SARS-CoV-2 proteins based on phosphomotifs and then to further capture their relevant host kinases based on their substrate motif and their activation status. By analyzing the association of the phosphosites in the human kinases perturbed by SARS-CoV-2 infection, the activation status of human kinases during infection was captured. Further, we have also explored the reported impact of various kinase inhibitors on SARS-CoV-2 infection. Incorporating these layers of information, we validated several kinase–substrate pairs using *in-silico* approaches, including MAP2K1 with ORF9b_S50, NSP2_Y124, NSP9_T77, NSP12_Y516, NSP13_Y582, N_S206, N_T198, and N_T296. This study not only expands the human SARS-CoV-2 viral proteome interactions but also provides a reference for applying this approach to other human pathogens.

## 2 Materials and methods

### 2.1 Selection of human kinase phosphomotifs

We retrieved kinases and their substrate motif sequences from the kinase-substrate target preference study by Poll et al., 2024 (Poll et al., 2024). Sugiyama et al., 2019 conducted an analysis that identified 175,574 potential direct kinase substrates and provided a comprehensive characterisation of substrate phosphorylation motif preference for 385 recombinant human protein kinases (Sugiyama et al., 2019). Poll et al., 2024 employed PTM-Logo software to generate sequence motif logos that visually depicted the substrate preferences of 384 recombinant human protein kinases, as developed by Sugiyama et al., 2019. PTM-Logo software was used to analyse 13-amino-acid centric sequences for each kinase, in accordance with the method outlined by Saethang et al., 2019 (Saethang et al., 2019). To generate reliable kinase substrate logos, a minimum of 30 target amino acid sequences were utilized. However, the number of input sequences required to determine kinase specificity may vary based on the strength of kinase-substrate interactions. Previous studies have reported that kinase preferences can often be identified with as few as 5 to 20 input sequences (Hornbeck et al., 2015). To ensure the appropriate selection of kinase substrate motif sequences for identifying potential substrates in SARS-CoV-2 viral proteins, specific parameters, along with particular inclusion and exclusion criteria, were considered. Among the 384 human substrate sequence motifs of recombinant protein kinases, only the 330 statistically significant kinase substrate motifs were selected and considered for the Kinase-Substrate motif pattern analysis in this study. 52 of the identified kinases were excluded from the study based on exclusion criteria, while seven were identified with low-intensity residues. Furthermore, 42 kinases with less than 30 protein kinase target sequences lacked consensus residues, while 3 kinases with statistically insignificant residues were excluded from the analysis. In addition to these criteria, the data underwent preprocessing to remove any sequences that were incomplete or ambiguous, ensuring the highest quality for motif identification. The Kinase-Substrate motif pattern analysis was performed using PTM-Logo with a motif length of 13 amino acids with a window size of -6/+6, along with Chi-squared filtering (alpha = 0.0001) to identify statistically significant motifs. The amino acid color-coding used in PTM-Logo was based on physicochemical properties, with hydrophobic residues (L, I, M, V, A) in green, and basic residues (K, R, H) in blue, providing insight into their potential interactions with kinase active sites (Poll et al., 2024).

### 2.2 Human kinome substrate phosphomotif analysis in SARS-CoV-2 proteins

The viral protein FASTA sequences of SARS-CoV-2 were retrieved from the NCBI and the UniProt databases (taxonomy ID: 2697049) (Al-Qaaneh et al., 2021). To identify potential host kinases for viral protein substrates, the kinase substrate motif sequences were searched against the FASTA sequences of 33 SARS-CoV-2 proteins. To explore the similarity or disparity of the viral phosphosite motifs across variants we have performed sequence alignment of 7,041 SARS-CoV-2 protein sequences in the UniProt database using Clustal Omega (Li, 2003). We have also evaluated the conservation of these motifs by comparing the amino acid sequences using a window size of -5 to +5 to identify substrate kinase matches within the sequences. Based on this analysis, conserved substrate motifs were identified and examined their association with kinase activity, focusing on their roles in viral replication and host-pathogen interactions.

### 2.3 SARS-CoV-2 induced alterations in the human cellular phosphoproteome

To identify host kinases modulated in SARS-CoV-2 infection, host phosphoproteomics data from various studies were obtained. Towards this, to compile human cellular global phosphoproteome datasets of SARS-CoV-2 infection, we screened the published literature using the PubMed search query “phosphoproteomics” AND “SARS-CoV-2”, and assembled Class I phosphosites (localization probability ≥ 75% or A-score >13). Each quantitative differential datasets comparing SARS-CoV-2 infection conditions against uninfected conditions were assembled. Differential datasets were assembled according to the criteria defined by the authors of each study (p-value <0.05; fold change, up-regulated as ≥ 1.3 and down-regulated ≤ as 0.76). A standardized format was implemented to define the biological and experimental conditions for each differential dataset. Individual phosphosites in each dataset were mapped to their corresponding UniProt accessions (downloaded in June, 2024) using our custom mapping tool to ensure consistent annotation (UniProt, Consortium, 2023).

### 2.4 Analysis of human kinase-viral protein interactions

We incorporated the human-viral protein-protein interaction (PPI) data for SARS-CoV-2 to evaluate the potential interactions between the predicted phosphorylated viral proteins and the kinases identified through the pattern search using human kinase substrate motif. The comprehensive PPI datasets were compiled from multiple databases of SARS-CoV-2 (Taxonomy ID: 2697049), including the latest versions from the following databases, HVIDB (Human-Virus Interaction DataBase) (Yang et al., 2021) on December 1, 2024; HVPPI (Human–Virus Protein-Protein Interaction database) (Li et al., 2022) on September 19, 2024; VirHostNet (Virus–Host Network) (Guirimand et al., 2015) on November 15, 2024; IntAct (Kerrien et al., 2012) on October 31, 2024; and BIOGRID (Oughtred et al., 2021) on September 9, 2024.

### 2.5 Investigation of regulatory phosphosites in kinases perturbed during SARS-CoV-2 infection

To investigate the role of predicted novel kinases in SARS-CoV-2 infection, an expression-based analysis was conducted using phosphoproteome datasets from SARS-CoV-2 infected human hosts. In order to examine the regulatory functions of phosphosites in kinases perturbed by SARS-CoV-2 infection, the activation and inhibition status of these phosphosites in the predicted kinases were analyzed using the curated regulatory site information from the PhosphoSitePlus database (Hornbeck et al., 2015).

### 2.6 Knowledge-based analysis of the human kinases and their association with SARS-CoV-2 proteins

An evidence-based analysis was conducted to assess whether the predicted kinase substrate motifs of SARS-CoV-2 viral proteins have been previously reported in the literature. Further, we also looked into whether the kinases selected based on expression analysis are experimentally validated for their association with viral protein. For this, experimentally validated data on viral protein phosphorylation sites, along with kinase and viral protein phosphorylation information, were collected from various databases, such as VPTMdb (Xiang et al., 2021), and from several other studies (Klann et al., 2020; Yaron et al., 2022; Bouhaddou et al., 2020; Hekman et al., 2020; Guo et al., 2022; Hekman et al., 2021; Davidson et al., 2020; Stukalov et al., 2021).

### 2.7 Structural analysis of Human kinase and SARS-CoV-2 protein

The Protein Data Bank (Berman et al., 2000) was utilized to obtain the 3D structure of human kinase MAP2K1 and SRPK1 (PDB ID: 4MNE – Crystal structure of the BRAF: MEK1complex) with a resolution of 2.85 Å (Haling et al., 2014), and the structure of SRPK1 (PDB ID: 5MXX – Crystal structure of human SR protein kinase 1 (SRPK1) in complex with compound 1) was resolved at 1.75 Å (Batson et al., 2017). SARS-CoV-2 viral protein structures were retrieved from PDB. Structures of Orf9b (PDB ID: 6Z4U – X-ray Crystallographic Structure of Orf9b from SARS-CoV-2) with a resolution of 1.95 Å, NSP2 (PDB ID: 7MSX – SARS-CoV-2 Nsp2) with a resolution of 3.15 Å (Gupta et al., 2021), NSP9 (PDB ID: 6WXD – SARS-CoV-2 Nsp9 RNA-replicase) with a resolution of 2.00 Å, NSP12 (PDB ID: 6NUR – SARS Coronavirus NSP12 bound to NSP7 and NSP8 co-factors) with a resolution of 3.10 Å (Kirchdoerfer and Ward, 2019), Nucleoprotein (PDB ID: 8FD5 - Nucleocapsid monomer structure from SARS-CoV-2) with a resolution of 4.57 Å (Kirchdoerfer and Ward, 2019), and NSP13 (PDB ID: 6ZSL - Crystal structure of the SARS-CoV-2 helicase at 1.94 Angstrom resolution) with a resolution of 1.94 Å (Newman et al., 2021) were selected. Using the protein preparation wizard, all the protein structures were optimized and energy minimized using opls4 (version of schrodinger 2024-4).

### 2.8 Molecular docking

Using the protein–protein docking of biologics (Navhaya et al., 2024) in Maestro v12.8, SARS-CoV-2 proteins ORF9b, NSP2, NSP9, NSP12, and NSP13 were selected as ligands against the human kinase MAP2K1, while the N protein was specifically docked with human kinase SRPK1 to assess whether the experimentally validated phosphorylation sites of the N protein interact with the human kinase SRPK1. Protein-protein docking was conducted by specifying the attraction and repulsion using the catalytic domain and phosphosite in respective to SARS-CoV-2 protein. The docked protein and ligand complex interaction was analyzed using the protein interaction analysis module. Thirty poses were generated for the complex and ranked based on PIPER cluster size, PIPER pose energy, and PIPER pose score, which evaluates receptor-ligand interactions and is efficiently computed using Fast Fourier Transforms (Mondol et al., 2023). The best-selected protein-protein docking pose (prot-prot-docking_2_pose_1) was characterized by its cluster size, PIPER pose energy, and PIPER pose score. The output complexes were carefully evaluated for binding at the active site, with particular attention given to the PIPER Pose Energy and cluster size. The distance between the kinase domain of MAP2K1, critical residues S218 and S222, and the viral protein phosphosites was calculated to evaluate their proximity and determine whether they could serve as substrates for MAP2K1. Additionally, the distance from the HRD region in MAP2K1, specifically for residues H188, R189, and A190, was analyzed to assess the potential interaction with MAP2K1. For the SRPK1–N protein complex, similar distance measurements were performed to evaluate interactions between the kinase domain region of SRPK1, particularly residues D497, L498, and G499, and the phosphosites of the N protein. These distances were measured using the Maestro v12.8 software.

## 3 Results

### 3.1 Identification of substrate phosphomotifs of human kinases in SARS-CoV-2 viral proteome

**Figure 1:**
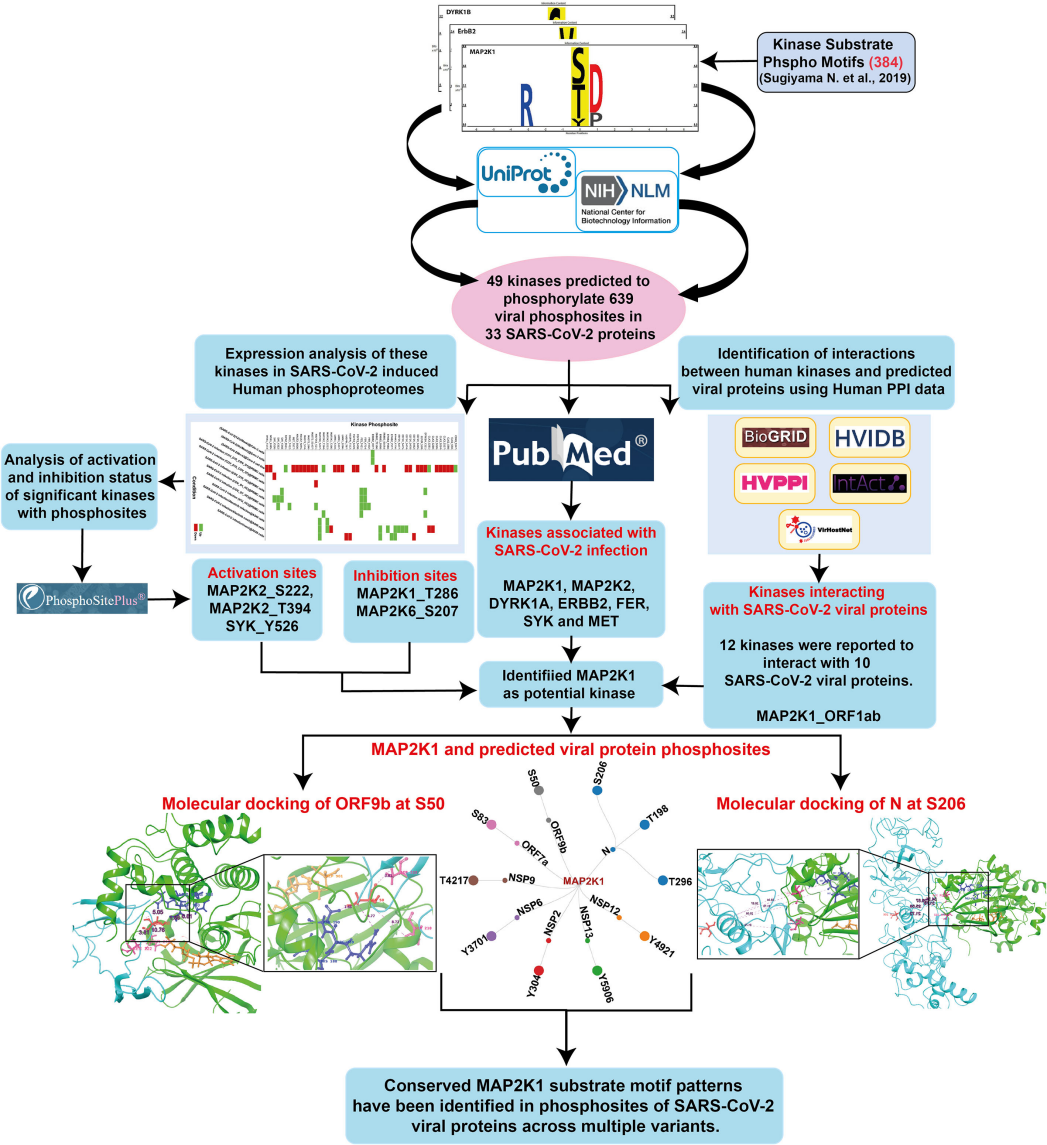
An integrative workflow for analyzing phosphomotifs, phosphoproteome, and interactome to identify potential human kinase substrates in SARS-CoV-2.

The human kinome–substrate motif pattern analysis resulted in the identification of 49 human kinases pertaining to 639 phosphosites across 33 SARS-CoV-2 viral proteins. Of these, 18 were classified as Serine/Threonine kinases, 21 were Tyrosine kinases, and 10 were dual-specificity kinases. This suggests that different host kinases have the potential to target distinct phosphorylation sites within viral proteins. These interactions within the host cells may be associated with regulatory processes involving multiple kinases that contribute to SARS-CoV-2 pathogenicity or immune evasion. The workflow of the analysis is given as **Figure 1** and the results of the data is given in the **Supplementary Table 1**.

### 3.2 Kinases modulated by SARS-CoV-2 infection based on host phosphoproteomes

**Figure 2:**
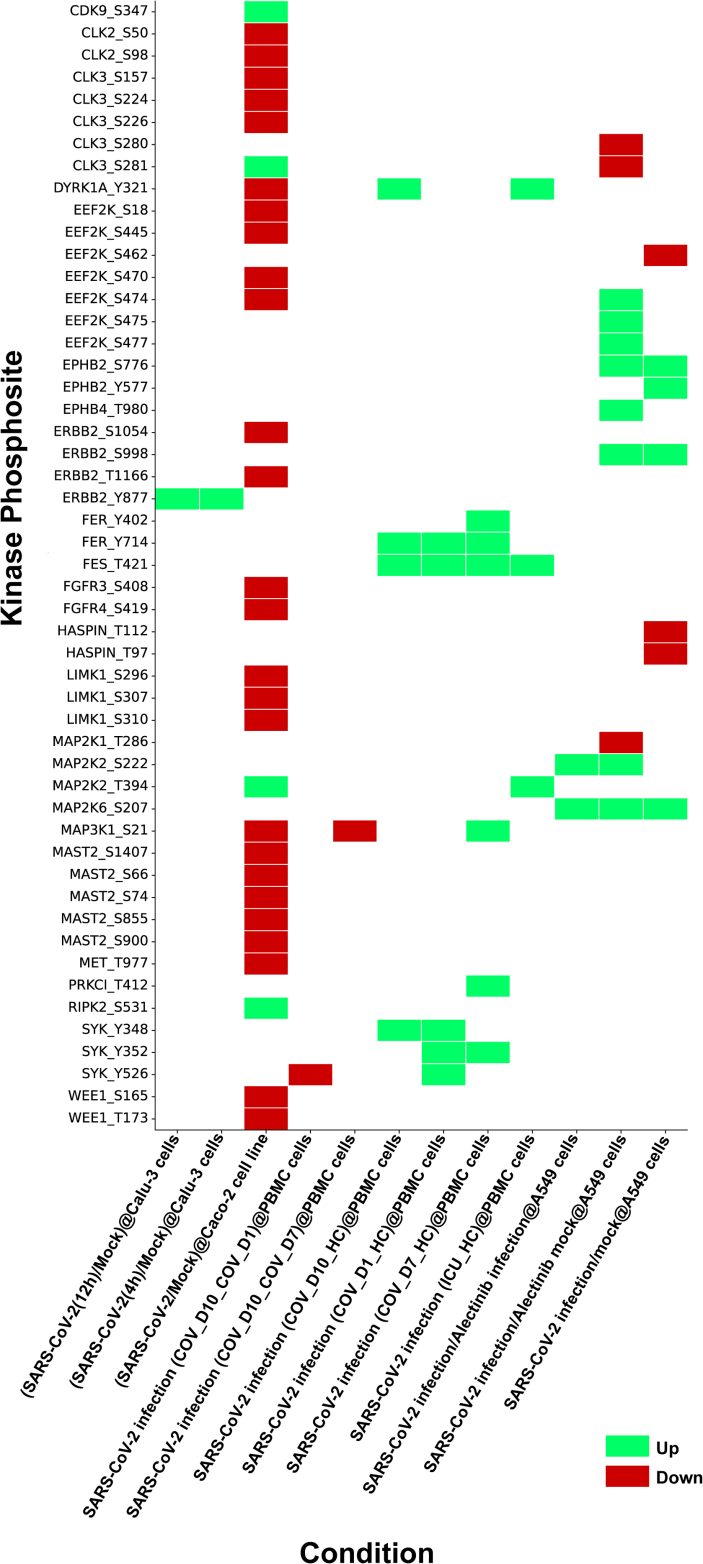
Heatmap depicting kinases identified through kinase substrate motif analysis and their expression profiles from SARS-CoV-2 phosphoproteome datasets compiled from multiple independent studies under various experimental conditions. The figure illustrates phosphosite specific regulation of these kinases during SARS-CoV-2 infection, with upregulated sites shown in green and downregulated sites in red.

Although human kinome-substrate motif analysis predicted 49 kinases, the host phosphoproteome of SARS-CoV-2 infected cells identified 24 of these kinases. There were 51 unique phosphorylation sites in these kinases that were perturbed by SARS-CoV-2 in multiple studies. These datasets were consolidated from 12 datasets corresponding from 5 distinct PMIDs. They were observed across multiple studies, including various host cell types and (ACE2+/expressed) cell line systems such as A549cells, Caco-2 cells, the PBMC (Peripheral Blood Mononuclear Cell) cell line, Calu-3 cells, and HEK293T cells. In this expression analysis, we pinpointed 15 kinases and their 23 phosphosites that were upregulated in 9 experimental conditions, and 15 kinases and their 33 phosphosites that were downregulated in 5 experimental conditions in the SARS-CoV-2 infected host phosphoproteome. The expression patterns of these kinases are depicted in the heatmap as represented in **Figure 2** and data is shown in **Supplementary Table 2**. Prior knowledge-based analysis suggests that certain kinases, such as MAP2K1, MAP2K2 (Mitogen-activated protein kinase 2), DYRK1A (Dual specificity tyrosine-phosphorylation-regulated kinase 1A), ERBB2 (Erb-B2 Receptor Tyrosine Kinase 2), FER, SYK (Spleen Tyrosine Kinase), and MET, may play a role in SARS-CoV-2 infection and pathogenesis. However, due to the limited and sometimes conflicting data available for some of these kinases, such as for DYRK1A, their precise roles in SARS-CoV-2 infection remain to be fully elucidated and require further investigation. This indicated that our downstream analysis showed promise in exploring human kinome-viral protein interactions (**Supplementary Table 3**).

### 3.3 Characterization of phosphosites in kinases perturbed by SARS-CoV-2

The phosphosites in 24 kinases perturbed by SARS-CoV-2 phosphoproteomes were analyzed for their association with the activation/inhibition status of kinase activity. We interpret these phosphorylation changes as part of a dynamic regulatory system in response to the infection. The upregulation of activation sites indicates enhanced kinase activity, while the downregulation of inhibitory sites suggests a disruption of negative regulation, likely contributing to the altered host signaling pathways during SARS-CoV-2 infection. We identified upregulation of the activation sites in several kinases, including MAP2K2_S222, MAP2K2_T394 and SYK_Y526 and the inhibition sites in kinases such as MAP2K1_T286 and MAP2K6_S207 were found downregulated. While our data strongly point to the functional involvement of these kinases, further experimental validation through direct assays or additional models is necessary to confirm their precise role. Thus, we propose these findings as potential directions for future research to further explore the mechanistic implications of these phosphorylation events in SARS-CoV-2 pathogenesis. Expression of these kinases and its corresponding viral sites were assessed within various SARS-CoV-2 human phosphoproteome datasets (**Supplementary Table 4**).

### 3.4 Comparative analysis of known and predicted phosphosites in SARS-CoV-2 proteins and their human kinases

**Figure 3:**
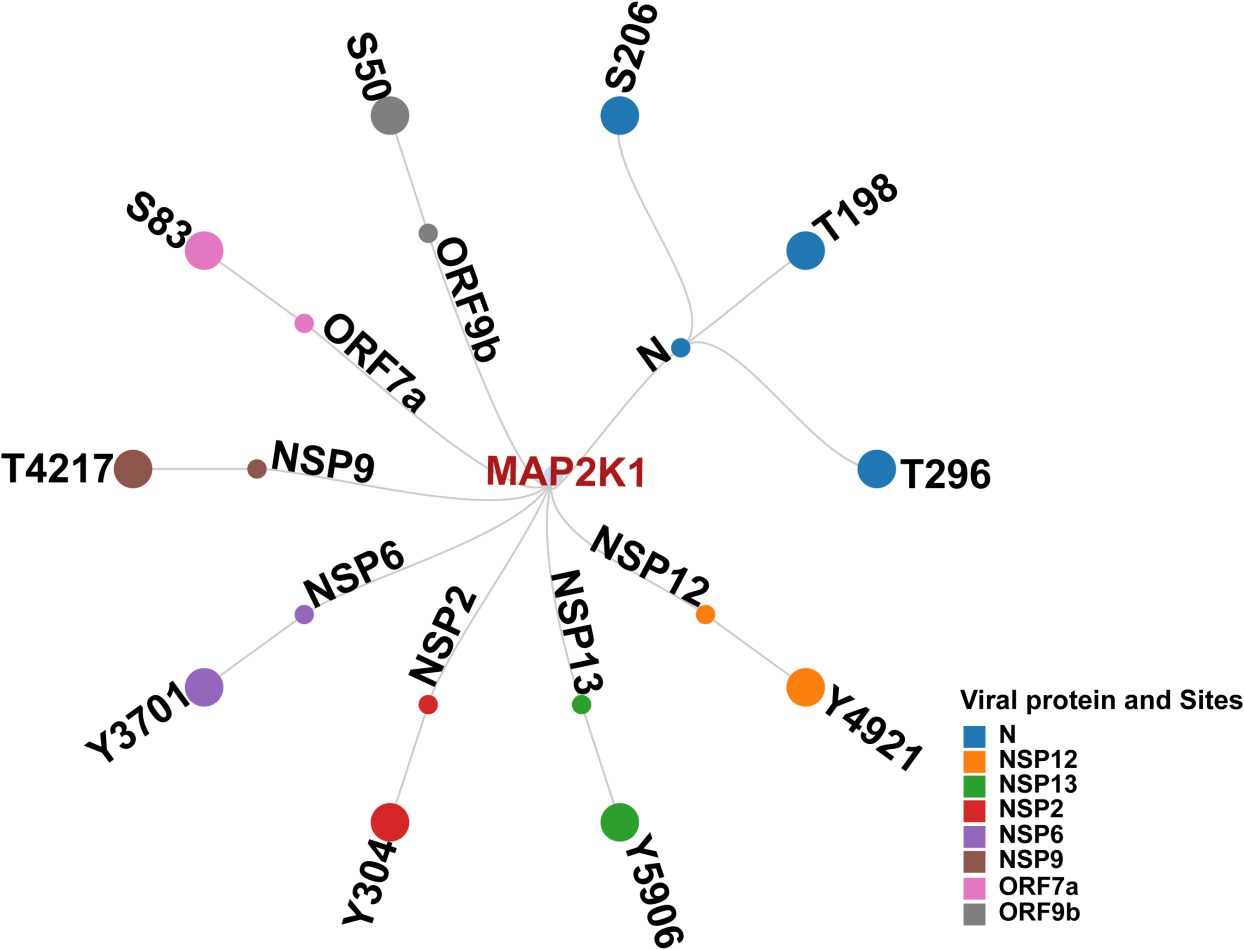
Network illustrating Human kinase MAP2K1, its predicted interacting viral proteins, and corresponding phosphosites.

We first examined phosphorylation sites in SARS-CoV-2 proteins reported in various studies. Distinct viral proteins and their phosphorylation sites that were experimentally reported/validated were curated from multiple studies. Together, 98 phosphosites in 11 SARS-CoV-2 proteins were identified to be reported (Hekman et al., 2021; Davidson et al., 2020; Stukalov et al., 2021; Xiang et al., 2021; Klann et al., 2020). This suggested that the phosphorylation in SARS-CoV-2 proteins is significant for establishing its infection. Furthermore, few host kinases were also found to be associated with the phosphorylation of specific viral proteins. These included SRPK1 (Serine–arginine protein kinase 1) (N_S206 and N_S188) (Yaron et al., 2022), PRKACA (cAMP-dependent protein kinase catalytic subunit alpha) (NSP13 _T198) (Bouhaddou et al., 2020), CDK2 (Cyclin-dependent kinase 2) (NSP12 _T20) (Guo et al., 2022), GSK3B (Glycogen synthase kinase-3 beta) (N_S176, N_S180, and N_T391) and CK2A1 (Casein kinase II subunit alpha) (N_ S23, N_S410, and N_S23) (Hekman et al., 2020). The experimentally validated phosphosites (S206 and T198) in Nucleocapsid (N), as well as S50 in ORF9b were found to be reported in multiple SARS-CoV-2 infection studies (Bouhaddou et al., 2020; Klann et al., 2020; Hekman et al., 2020; Davidson et al., 2020; Stukalov et al., 2021). These were also predicted kinase motif phosphosites of human kinases. Our phosphomotif pattern analysis identified MAP2K1, a kinase for these phosphosites known to be perturbed by SARS-CoV-2 infection, which is in consensus with studies using MAP2K1 inhibition (Zhang et al., 2021; Yaron et al., 2022; Xie et al., 2022; Weckbach et al., 2022; Mizutani et al., 2004) (**Figure 3**).

### 3.5 Analysis of human kinome-viral protein interactions

We analyzed the interaction data of the 49 kinases identified in the motif analysis with human-viral protein-protein interactions to determine if any of these predicted kinases interact with SARS-CoV-2 viral proteins. Based on the interaction data from various Human-Viral Protein-Protein PPI databases, 12 kinases were reported to interact with 10 SARS-CoV-2 viral proteins. The expression of seven of these kinases and their 24 phosphorylation sites were identified across five SARS-CoV-2 human host phosphoproteomics datasets from three different studies (**Supplementary Table 5**). Among them, MAP2K1 and ERBB2 kinases were identified from previous studies to play a significant role in SARS-CoV-2 infection and is exemplified by the impact on SARS-CoV-2 infection upon their inhibition (Weckbach et al., 2022; Zhang et al., 2021; Schreiber et al., 2022; Xie et al., 2022; Saul et al., 2023). On the basis of predicted viral phosphosites identified using the substrate motif search, five phosphorylation sites in N proteins S206 (Hekman et al., 2021; Davidson et al., 2020; Xiang et al., 2021), T198 (Hekman et al., 2021; Xiang et al., 2021), T391 (Xiang et al., 2021; Davidson et al., 2020; Stukalov et al., 2021), S410 (Hekman et al., 2021; Stukalov et al., 2021), and S310 (Stukalov et al., 2021) were previously identified in various studies involving SARS-CoV-2 infection. Beyond this, based on motif analysis, we also predicted 107 phosphorylation sites in 10 SARS-CoV-2 proteins with the potential for seven kinases to phosphorylate them.

### 3.6 SARS-CoV-2 variants showed conservation of substrate motifs in MAP2K1

**Figure 4:**
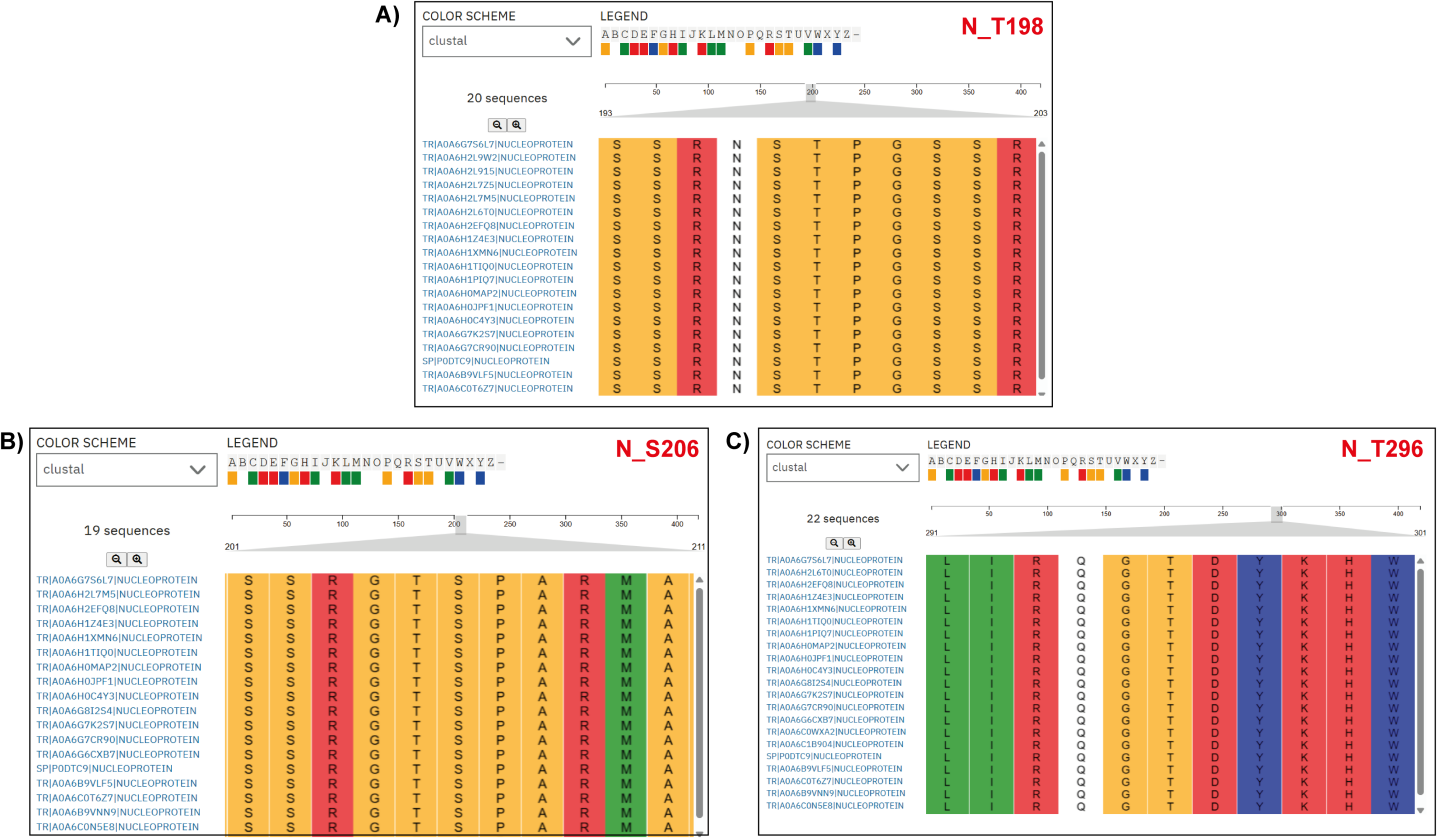
Multiple Sequence Alignment (MSA) of nucleoprotein (N protein) sequences from various SARS-CoV-2 variants. The alignment highlights a specific region, emphasizing the conservation of the MAP2K1 kinase substrate motif patterns across SARS-CoV-2 variant nucleoprotein sequences. The accession labels in the figure correspond to the SARS-CoV-2 variant types. **(A)** N_T198 **(B)** N_S206, and **(C)** N_T296 are the predicted sites across the SARS-CoV-2 variant nucleoprotein sequences.

To examine MAP2K1 viral protein substrates, we mapped kinase-substrate motif sequences from Poll et al. (2024) against viral protein FASTA sequences from different SARS-CoV-2 variants (Poll et al., 2024). From the analysis we have identified completely conserved window sequences and sequences with a partial match (i.e., conserved “R&D/P motif”, characterized by an R at position –3 and either a D or P at +1 relative to the modification site, within a –5 to +5 sequence window). For the NSP9 protein at phosphosite T4217, total of 3633 variant NSP9 protein sequences were analyzed, with 3364 (92.6%) exact motif matches to the MAP2K1 kinase substrate motifs and six conserved R&D/P motif patterns. Similarly, at position Y304 in NSP2, 3659 variant protein sequences were analyzed, with 3234 (88.4%) exact motif matches and 39 conserved R&D/P motifs patterns. For NSP13 at position Y5906, 1994 sequences were analyzed, showing, 1846 (92.6%) exact motif matches, and 20 conserved motifs patterns. In NSP12 at position Y4908, 1863sequences showed 1,859 (99.8%) exact motif matches, along with three conserved motifs patterns. Within the N protein, 499 variant sequences were analyzed for each site. At position T198, 171 sequences (34.3%) showed exact motif matches and 184 conserved motifs patterns. At position S206 in N, with 257 (51.5%) exact motif matches and 86 conserved motifs patterns. At position T296, with 459 (92.0%) exact motifs matches, and seven conserved motifs patterns. A complete conservation of the MAP2K1 substrate motif was observed at position S50 of ORF9b (100%) in the only available sequence (**Supplementary Table 6**) (**Figure 4**). Most MAP2K1 substrate motifs were identified through exact matches or conserved patterns, indicating strong conservation at multiple phosphorylation sites across SARS-CoV-2 variants, with particularly high conservation observed at specific phosphosites.

### 3.7 Molecular docking results of MAP2K1 kinase and its SARS-CoV-2 substrate protein phosphosites

**Figure 5:**
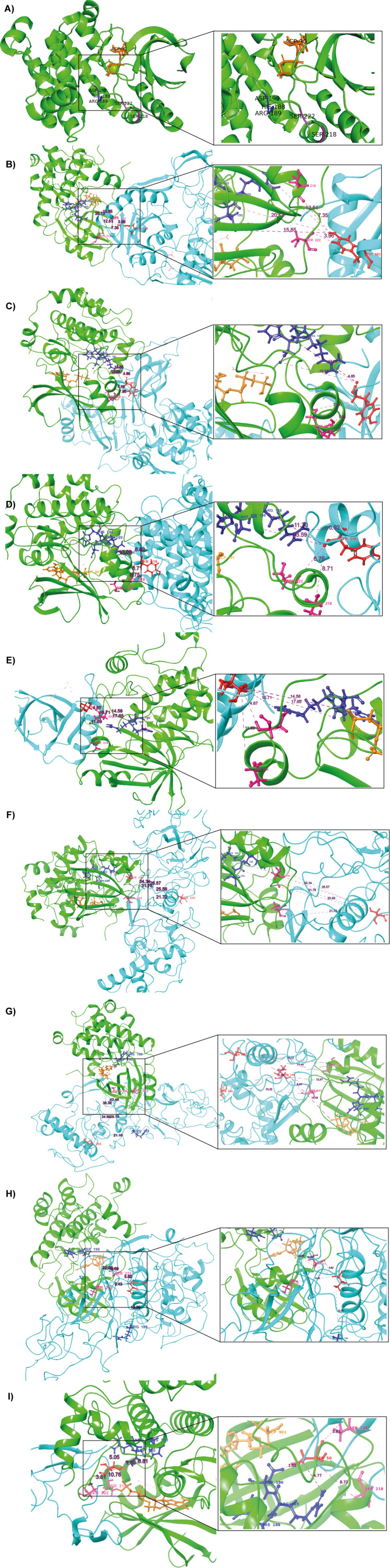
Docking results of MAP2K1 with predicted phosphorylation sites on SARS-CoV-2 viral proteins. **(A)** The 3D structure of the MAP2K1 protein (PDB ID: 4MNE), highlighting key residues within the catalytic domain. Green colour indicates the kinase (MAP2K1), blue colour represents the viral proteins, and red colour marks the predicted phosphorylation sites on SARS-CoV-2 viral proteins. Dark blue colour highlights the HRD and DFG regions, which are critical for kinase activity. Purple colour indicates the residues of the catalytic loop, specifically Ser218 and Ser222. **(B)** MAP2K1 and SARS-CoV-2 NSP13 predicted phosphorylation site at T582. **(C)** MAP2K1 and SARS-CoV-2 NSP2 predicted phosphorylation site at Y124. **(D)** MAP2K1 and SARS-CoV-2 NSP12 predicted phosphorylation site at T516. **(E)** MAP2K1 and SARS-CoV-2 NSP9 predicted phosphorylation site at T77. **(F)** MAP2K1 and SARS-CoV-2 N protein predicted phosphorylation site at S206. **(G)** MAP2K1 and SARS-CoV-2 N protein predicted phosphorylation site at T296. **(H)** MAP2K1 and SARS-CoV-2 N protein predicted phosphorylation site at T198. **(I)** MAP2K1 and SARS-CoV-2 ORF9b predicted phosphorylation site at S50.

Protein–protein docking using biologics (Navhaya et al., 2024) generated 30 docking poses as output. Top scoring pose were selected for analysis with a PIPER pose energy of -511.525, a cluster size of 146, and a PIPER pose score of 116.246 for the complex MAP2K1 and the ORF9b viral protein and its viral protein phosphosite S50. With a PIPER pose energy of -643.932, a cluster size of 176, and a PIPER pose score of -141.954, the viral phosphosite Y582, which is the top-scoring pose for the complex MAP2K1 and the viral protein NSP13, was identified. The complex MAP2K1 and the viral protein NSP12 and the viral protein phosphosite Y516, with a PIPER pose energy of -619.618, a cluster size of 147, and a PIPER pose score of -64.216 respectively. The top-scoring position for the complex MAP2K1 and the viral protein NSP9 in the viral phosphosite was selected for analysis based on a PIPER pose energy of -443.695, a cluster size of 140, and a PIPER pose score of -82.298. A PIPER pose energy of -643.932, a cluster size of 176, and a PIPER pose score of -141.954 were used to select for analysis the top-scoring pose for the complex MAP2K1 and the viral protein NSP2 and its viral protein phosphosite Y124. The top-scoring pose for the complex MAP2K1 and its Nucleoprotein and its viral protein phosphosites T198, S206, and T296, was selected for analysis with a PIPER pose energy of -511.525, a cluster size of 146, and a PIPER pose score of 116.246. The docking interactions between MAP2K1 and the viral proteins NSP13, NSP2, NSP12, NSP9, ORF9b, and N protein revealed stable binding, with notable interactions at specific phosphorylation sites. MAP2K1 was identified as a kinase that can potentially phosphorylate these viral proteins at the sites, as indicated by the favorable docking poses. The results highlight the strength of interactions between MAP2K1 and the viral proteins, suggesting that these complexes are likely to exhibit significant binding affinity and structural stability, particularly at the active sites. These findings suggest that MAP2K1 may play a role in modulating the functions of these viral proteins by phosphorylating them. The docking results of MAP2K1 are provided in **Supplementary Table 7** and represented in **Figure 5**. Docking analysis of SRPK1 with the SARS-CoV-2 N protein binding at the phosphosites T198 and S206, with a PIPER pose energy of -1623.654, a cluster size of 129, and a PIPER pose score of -35.874. This suggests that SRPK1 may also act as a host kinase capable of targeting the N protein for phosphorylation. The docking results of SRPK1 are given in **Supplementary Table 8** and represented in **Supplementary Figure 1**.

### 3.8 Inferring potential kinase-substrate relationship between MAP2K1 and SARS-CoV-2 proteins

The phosphorylation site ORF9b_S50 was situated at the catalytic cleft of MAP2K1, specifically, 8.72 Å from the active site residue S218 and 3.81 Å from S222. Similarly, NSP2_Y124 is positioned 8.31 Å from S218 and 6.34 Å from S222, while NSP9_T77 is found at distances of 10.83 Å and 4.49 Å from S218 and S222, respectively. The site NSP12_Y516 is located 6.99 Å from S218 and 6.75 Å from S222, and NSP13_Y582 is 7.35 Å and 2.62 Å away from S218 and S222, respectively. For the N protein, the phosphorylation site N_S206 is at distances of 25.57 Å from S218 and 21.64 Å from S222, while N_T198 is 5.62 Å from S222, and N_T296 is located 31.69 Å from S218. The distance analysis reveals that the phosphorylation sites of viral proteins are located at varying distances from the catalytic cleft residues S218 and S222 of MAP2K1. The distance between the HRD region of the kinase domain and the predicted phosphorylation sites in the viral proteins in the SARS-CoV-2 are given in **Supplementary Table 9**. Correspondingly, the distances from the DLG motif region of SRPK1 to the viral N protein phosphosites are presented in **Supplementary Table 10**.

## 4 Discussion

The identification of host-viral protein-protein interactions is a major challenge for the scientific community. Human kinases are established to play a critical role in regulating host-pathogen interactions, particularly in the context of viral infections such as in SARS-CoV-2 infection. With phosphoproteomics emerging as an efficient tool for identifying phosphosites in host and viral proteins, phosphosites that are detected together offers the opportunity to explore kinase-substrate interactions between them. The analysis of host kinase substrate motifs has provided crucial insights into the specificity and regulation of kinase signaling pathways. By identifying conserved motifs, we can focus on specific kinases and explore the mechanisms by which kinases recognize and phosphorylate their substrates. This becomes critical in the context of host-pathogen interactions wherein the therapeutic approaches are often limited. In this study, we devised a strategy that entails human phosphomotif search against pathogen proteins and coupled them to the phosphoproteome and interactome datasets to predict potential kinase-substrate interactions between host and pathogen. More specifically, it provides a predictive framework to identify specific phosphosites in kinases that are associated with its kinase activity and phosphorylate substrate proteins.

Undertaking this approach to SARS-CoV-2 viral proteome, our motif analysis identifies 24 human kinases, with potential role in SARS-CoV-2 infection at the phosphoproteome level. Prior studies referred below highlighted the association of some of these kinases in host-pathogen interactions and their potential as therapeutic targets in SARS-CoV-2 infection. MAP2K1 and MAP2K2, integral to the MAPK/ERK signaling pathway, have been shown to regulate inflammation and interferon responses, with MAP2K2 deactivation promoting the resolution of acute lung injury (ALI) (Sarkar et al., 2022). In SARS-CoV-2 patients, MAP2K1 is significantly upregulated, and MEK inhibitors, such as selumetinib, have been shown to reduce lung damage and improve survival in infected animal models, suggesting that modulation of this pathway may provide therapeutic benefits, potentially through both direct and indirect mechanisms (Weckbach et al., 2022; Xie et al., 2022; Zhang et al., 2021). DYRK1A has been identified as a regulator of ACE2 (Angiotensin-converting enzyme 2) and DPP4 (Dipeptidyl Peptidase 4) expression, facilitating viral entry through a kinase-independent mechanism, with knockout studies further confirming its role in SARS-CoV-2 infectivity (Strine et al., 2023; Mao et al., 2024). The ErbB family, particularly ErbB2, has been shown to mediate viral internalization and ACE2 regulation, with inhibitors like lapatinib demonstrating antiviral potential (Saul et al., 2023). Additionally, the FER tyrosine kinase has been directly inhibited by SARS-CoV-2 ORF6, indicating evolutionary adaptations in viral proteins (Granata et al., 2023). SYK kinase has been implicated in severe COVID-19 immunopathogenesis, contributing to proinflammatory responses and NET formation, with inhibitors like fostamatinib reducing inflammation and thromboinflammation (Wigerblad et al., 2023; Noda, 1999; Theobald et al., 2022). Furthermore, MET inhibition by capmatinib has shown broad-spectrum antiviral activity against coronaviruses by disrupting replication processes in a MET-independent manner (Sugiyama et al., 2021). Collectively, these studies underscore the significance of kinases in SARS-CoV-2 infection and their potential as therapeutic targets to mitigate disease progression and severity.

In this regard, the method we have adopted demonstrated the potential for the identification of kinase-substrate interactions. Subsequently, based on *in-silico* analysis, one of the human kinase, MAP2K1, was identified as a putative kinase that can phosphorylate ORF9b, N protein, NSPs, including NSP12, NSP13, NSP2, NSP6 and NSP9. MAP2K1, a mitogen-activated protein kinase, plays a crucial role in the Raf/MEK/ERK signaling pathway, which is transiently activated during the early stages of SARS-CoV-2 infection. Studies have shown that ATR-002, a selective inhibitor targeting MAP2K1/2 kinases, exhibits potent anti-SARS-CoV-2 activity in both cell lines and primary epithelial cell cultures (Schreiber et al., 2022). These findings highlight the potential of MAP2K1/2 inhibition as a therapeutic strategy against SARS-CoV-2 infection (Schreiber et al., 2022). The docking results show that each viral protein forms stable interactions with MAP2K1, indicating that MAP2K1 could play a role in modulating the function of these viral proteins. This highlights the potential significance of MAP2K1 in influencing SARS-CoV-2’s ability to interact with host cell machinery, which could be further explored for therapeutic targeting.

The interactions between SARS-CoV-2 proteins and host signaling pathways reveal critical mechanisms by which the virus manipulates host cellular processes to establish infection and evade immune responses (Rex et al., 2021) and further, long-term complications (Mahin et al., 2024). ORF9b, an accessory protein of SARS-CoV-2, plays a pivotal role in host-virus interactions. It targets TOM70, a component of the mitochondrial translocase of the outer membrane complex, disrupting mitochondrial antiviral signaling and enabling the virus to evade the host immune response (Ayinde et al., 2022). Furthermore, it inhibits the K63-linked polyubiquitination of NEMO (NF-kappa-B essential modulator), a key regulator of NF-κB signaling, thereby blocking the activation and nuclear translocation of NF-κB. This inhibition suppresses the production of proinflammatory cytokines, further aiding immune evasion (Wu et al., 2021). Notably, this protein has been identified with S50 as a phosphorylation site in multiple studies (Bouhaddou et al., 2020; Klann et al., 2020; Stukalov et al., 2021). The N protein, a structural component of SARS-CoV-2, plays a crucial role in viral replication and assembly. Emerging evidence highlights its multifunctional nature, emphasizing significant contribution to the pathogenesis of COVID-19 and modulation of the antiviral immunity. Moreover, nuclear factor-κB (NF-κB) and mitogen-activated protein kinase (MAPK) signaling pathways have been demonstrated to be activated by SARS-CoV-2 infection, highlighting critical role of the N protein in promoting these processes (Yu et al., 2023). Experimentally validated phosphosites of the N protein, S206 and T198, have been identified in multiple studies (Bouhaddou et al., 2020; Klann et al., 2020; Hekman et al., 2020; Davidson et al., 2020). Using phosphomotif pattern analysis, we predicted MAP2K1 as the upstream kinase responsible for phosphorylating the sites in both ORF9b (S50) and the N protein (S206 and T198) in SARS-CoV-2. Given these findings, we propose that MAP2K1 may phosphorylate S50 in ORF9b and S206 and T198 in the N protein during SARS-CoV-2 infection.

Integrating human protein–protein interaction data from Liu et al. (2021) with our phosphomotif-based predictions, we consistently identified MAP2K1 as a key host kinase interacting with and potentially targeting the SARS-CoV-2 ORF1ab protein (Liu et al., 2021). ORF1ab, the largest open reading frame (ORF) in the SARS-CoV-2 genome, is a major source of T-cell epitopes. As the first protein translated by the infected cell, ORF1ab represents a key target for early T-cell responses (Gustiananda et al., 2021). ORF1ab of the SARS-CoV-2 genome is processed into 15 non-structural proteins (NSPs) by proteases, with each NSP playing a specific role in the virus’s life cycle and pathogenicity (Raj, 2021). NSP2, another viral protein, suppresses IFN-β (interferon-beta) production by hijacking the GIGYF2/4EHP complex, interfering with the translation of Ifnb1 mRNA (Xu et al., 2022). This highlights several functions of NSP2 in immune evasion by allowing the virus to suppress the host’s innate immune response. On the other hand, NSP9 targets TBK1 (TANK-binding kinase 1), a crucial regulator of interferon signaling, to positively regulate the generation of cytokines (Zhang et al., 2023). The intricate relationships between viral proteins and host immunological pathways are highlighted by this dual function in immune regulation. NSP13, the helicase of SARS-CoV-2, exhibits catalytic functions dependent on Mg^2+^ concentrations, which regulate ATP hydrolysis, duplex unwinding, and RNA-protein remodeling processes crucial for viral replication and proofreading (Sommers et al., 2023). Its nucleotide-binding site and nucleic acid binding channel facilitate unwinding of double-stranded RNA or DNA, a vital function in viral replication (Soper et al., 2024). These insights into NSP13’s biochemical mechanisms provide a foundation for targeting its catalytic activity in therapeutic strategies. NSP12, a key component of the SARS-CoV-2 replication and transcription machinery, exhibits limited activity on its own. Its functionality is significantly enhanced by the presence of accessory proteins NSP7 and NSP8, which collectively achieve the highest activity rate. However, these accessory proteins alone are insufficient for the optimal functioning of NSP12, highlighting the intricate interplay required for efficient viral replication (Kakavandi et al., 2023). Previous studies have shown that SARS-CoV-2 proteins, including NSP2, NSP9, NSP13, and NSP12, manipulate host signaling pathways and cellular processes to facilitate viral replication, immune evasion, and pathogenesis. Building upon these findings, our docking analysis revealed a strong interaction between MAP2K1 and several SARS-CoV-2 viral proteins, with a particularly notable interaction observed with ORF1ab. This supports and extends prior observations, suggesting a potential mechanistic role for MAP2K1 in SARS-CoV-2 mediated host modulation. The inhibition of MAP2K1 and its subsequent impact on SARS-CoV-2 infectivity further demonstrates the potential role of MAP2K1 in modulating viral protein functions. Using phosphomotif pattern analysis, MAP2K1 was identified as the upstream kinase responsible for phosphorylating specific sites on SARS-CoV-2 viral proteins. Based on existing studies, MAP2K1 plays a pivotal role in regulating key processes during SARS-CoV-2 infection, as evidenced by its interaction with viral proteins, including ORF1ab (Liu et al., 2021). The ORF1ab of the SARS-CoV-2 genome is processed into 15 non-structural proteins (NSPs) by proteases, with each NSP playing a specific role in the virus’s life cycle and pathogenicity. Through phosphomotif pattern analysis, we predicted MAP2K1 as the upstream kinase responsible for phosphorylating various NSPs, including NSP12 at Y4921, NSP13 at Y5906, NSP2 at Y304, NSP6 at Y3701, and NSP9 at T4217, all of which are encoded by ORF1ab. These findings suggest that MAP2K1 may play a crucial role in modulating viral protein activity during SARS-CoV-2 infection by phosphorylating key NSPs. This phosphorylation could influence viral replication and immune evasion mechanisms, highlighting MAP2K1 as a potential therapeutic target for disrupting viral pathogenesis.

The analysis of MAP2K1 kinase substrate patterns across various SARS-CoV-2 viral proteins offers valuable insights into the conservation of phosphorylation sites and their potential biological roles. Many viral proteins, including NSP9, NSP2, and NSP13, show a high degree of conservation at specific phosphorylation sites, indicating that these sites are critical for regulating protein activity, stability, and interactions. The overall high conservation of these phosphosites suggests their fundamental role in the viral life cycle, particularly in mediating interactions between viral proteins and host cell signaling pathways. These findings enhance our understanding of the molecular mechanisms driving SARS-CoV-2 infectivity and highlight promising therapeutic targets for disrupting key phosphorylation events, offering a potential strategy for combating the virus.

The distance-based analysis of SARS-CoV-2 viral protein phosphorylation sites with MAP2K1’s catalytic residues (S218 and S222) and the HRD region highlights potential interactions that could influence viral protein regulation in pathogenesis of SARS-CoV-2 infection. Among the analyzed sites, ORF9b_S50 and NSP13_Y582 exhibit the strongest proximity to MAP2K1 catalytic residues and the HRD region, making them the most likely targets for direct phosphorylation. In addition, nucleoprotein and its viral protein phosphosites T198, S206, and T296, along with NSP2_Y124, NSP9_T77, and NSP12_Y516, show proximity to MAP2K1’s catalytic residues and the HRD motif, positioning them as potential candidates for phosphorylation. These results highlight MAP2K1’s significance in host-virus interactions and suggest that it may be involved in modifying SARS-CoV-2 protein activities.

SRPK1/2 has been experimentally shown in multiple studies to phosphorylate sites on the SARS-CoV-2 Nucleocapsid (N) protein (Yaron et al., 2022; Wu et al., 2023; Savastano et al., 2020). For this study, we selected SRPK1 as a model kinase because its biological interactions with the SARS-CoV-2 Nucleocapsid (N) protein have been experimentally validated. Our docking analysis of SRPK1 with the Nucleocapsid protein revealed a cluster size of 129 Å, a PIPER docking score of -1623.65 kcal/mol, and a PIPER pose energy of -35.874 kcal/mol. Upon further examination of the interaction interface, we observed that the kinase domain of SRPK1 was located at a considerable distance from key phosphorylation residues, specifically S206 and S222, on the Nucleocapsid protein. In comparison, docking analysis of MAP2K1 showed a larger cluster size of 146, indicating a more favorable interaction with the Nucleocapsid protein. MAP2K1’s interaction demonstrated a docking score of -511.525 kcal/mol and significant proximity to residues T198, S206, and T296. Notably, the catalytic residues of MAP2K1 (S218 and S222) were within 21.72 Å and 5.62 Å of N_S206 and N_T198, respectively, whereas SRPK1 domain regions were consistently over 35 Å away from these sites. These results suggest that MAP2K1 has a stronger potential to interact with and possibly phosphorylate the nucleocapsid protein at critical sites, indicating its higher efficacy compared to SRPK1.

Although there have been advancements in understanding viral-host interactions, the precise host kinases responsible for phosphorylating viral proteins are still not well understood. Developing computational approaches to identify phosphorylation sites and their associated host kinases can help enhance our understanding of viral protein regulation and uncover potential therapeutic targets. Most of the phosphosite patterns of viral proteins are conserved across variants, suggesting that these conserved sites may play an essential role in regulation of viral proteins. This observation indicates that viral proteins or viruses exploit the host kinase regulatory network or hijack it indirectly for their replication or other functions. Evaluation of the function of phosphorylation sites in SARS-CoV-2 viral proteins induced by human kinases will enhance our understanding of the mechanisms underlying viral invasion and pathogenesis. These insights will be helpful in advancing antiviral drug discovery, enabling precision vaccine design, and developing strategies to combat future variants of the virus.

## 5 Conclusions

Identifying phosphorylation modification sites in viral proteins such as in SARS-CoV-2 is essential for developing novel therapeutic strategies that can enhance global healthcare. Bioinformatics research has revealed significant alterations in protein phosphorylation during infection, but current literature provides limited insights into predicting these sites. Although inhibition of both MAP2K1/2 led to a reduction in SARS-CoV-2 infection that suggested their crucial role in the viral infection process, the potential kinase-substrate relationships with viral proteins are not yet established. As an outcome of our approach, we predict the potential of MAP2K1/2 as a kinase capable of phosphorylating SARS-CoV-2 viral proteins. Combining structural analysis of kinases with functional studies of phosphorylation sites in target viral proteins might help to explore relevant host-pathogen interactions. As a model, in this study, we provide a robust approach to identify kinases of respective phosphorylation sites in SARS-CoV-2 proteins, offering insights into the post-translational regulation of viral proteins by host kinases. The integration of computational prediction and experimental validation ensures a high degree of reliability in mapping kinase-substrate interactions for further validation, which could be pivotal for developing targeted antiviral therapies in viruses with limited therapeutic interventions.

## Data Availability

The datasets presented in this study can be found in online repositories. The names of the repository/repositories and accession number(s) can be found in the article/**Supplementary Material**.

## Supplementary material

Supplementary Figure 1 Docking results of SRPK1 with experimentally validated phosphorylation sites on the N viral protein at T198 and S206.

Supplementary Table 1 List of predicted kinases, viral proteins, and their corresponding viral phosphosites.

Supplementary Table 2 The table highlights phosphosite-specific regulation (Upregulated or Downregulated) of kinases during SARS-CoV-2 infection, as reported in multiple independent studies in different experimental condition. The kinases listed were predicted through kinase substrate motif analysis.

Supplementary Table 3 List of identified kinases derived from interaction data across various human-viral protein-protein interaction (PPI) databases, along with their expression validated at the phosphoproteome level in SARS-CoV-2 infection studies.

Supplementary Table 4 List of kinases identified in human cellular SARS-CoV-2 phosphoproteome studies along with their regulatory phosphorylation sites.

Supplementary Table 5 Expression of seven kinases and their 24 regulatory phosphorylation sites identified across five SARS-CoV-2 human host phosphoproteomics datasets from three independent studies.

Supplementary Table 6 The table summarizes the conservation of MAP2K1 phosphorylation sites identified on SARS-CoV-2 viral proteins across multiple variant sequences. It details the total number of MAP2K1 kinase substrate motifs conserved in viral protein sequences from different SARS-CoV-2 variants.

Supplementary Table 7 This table presents the molecular docking results of the human kinase MAP2K1 with selected SARS-CoV-2 viral proteins and their predicted phosphorylation sites. For each complex, the table includes the name of the protein–protein complex, the PDB ID used for MAP2K1, the specific phosphosite predicted on the viral protein, the PIPER pose energy, cluster size, and the PIPER pose score.

Supplementary Table 8 This table presents the molecular docking results of the human kinase SRPK1 with selected SARS-CoV-2 viral proteins and their predicted phosphorylation sites. For each complex, the table includes the name of the protein–protein complex, the PDB ID used for SRPK1, the specific phosphosite predicted on the viral protein, the PIPER pose energy, cluster size, and the PIPER pose score.

Supplementary Table 9 This table presents the distance analysis between predicted phosphorylation sites on SARS-CoV-2 viral proteins and the kinase domain of MAP2K1 (PDB ID: 4MNE). The kinase domain comprises key functional residues, including the HRD motif and the activation loop phosphorylation sites S218 and S222. Distances are measured from these critical residues to the corresponding viral protein phosphosites.

Supplementary Table 10 This table presents the distance analysis of phosphorylation sites of SRPK1 on viral N protein phosphosites, distances from the DLG motif region of SRPK1.

## Glossary

SARS-CoV-2: Severe Acute Respiratory Syndrome Coronavirus 2
SARS-CoV: Severe Acute Respiratory Syndrome Coronavirus
MERS-CoV: Middle East respiratory syndrome coronavirus
N protein: Nucleocapsid protein
MAP2K1: Mitogen-activated protein kinase 1
ORFs: Open reading frames
PLpro: Papain-like protease
Mpro: Main protease
Nsps: Nonstructural proteins
S protein: Spike protein
M protein: Membrane protein
E protein: Envelope protein
VOCs: Variants of concern
S: Serine
T: Threonine
Y: Tyrosine
PPI: Protein-protein interaction
HVIDB: Human-Virus Interaction DataBase
HVPPI: Human–Virus Protein-Protein Interaction database
VirHostNet: Virus–Host Network
BIOGRID: Biological General Repository for Interaction Datasets
PBMC: Peripheral Blood Mononuclear Cell
MAP2K2: Mitogen-activated protein kinase 2
DYRK1A: Dual specificity tyrosine-phosphorylation-regulated kinase 1A
ERBB2: Erb-B2 Receptor Tyrosine Kinase 2
SYK: Spleen Tyrosine Kinase
SRPK1: Serine–arginine protein kinase 1
PRKACA: cAMP-dependent protein kinase catalytic subunit alpha
CDK2: Cyclin-dependent kinase 2
GSK3B: Glycogen synthase kinase-3 beta
CK2A1: Casein kinase II subunit alpha
ACE2: Angiotensin-converting enzyme 2
DPP4: Dipeptidyl Peptidase 4
NEMO: NF-kappa-B essential modulator
TBK1: TANK-binding kinase 1
DYRK1A: Dual specificity tyrosine-phosphorylation-regulated kinase 1A.
