# Supplementary figures and images for: Analysis of phosphomotifs coupled to phosphoproteome and interactome unveils potential human kinase substrate proteins in SARS-CoV-2
Source: Front Cell Infect Microbiol. 2025 Jul 9;15:1554760. doi: 10.3389/fcimb.2025.1554760 (PMC12283625; doi:10.3389/fcimb.2025.1554760)

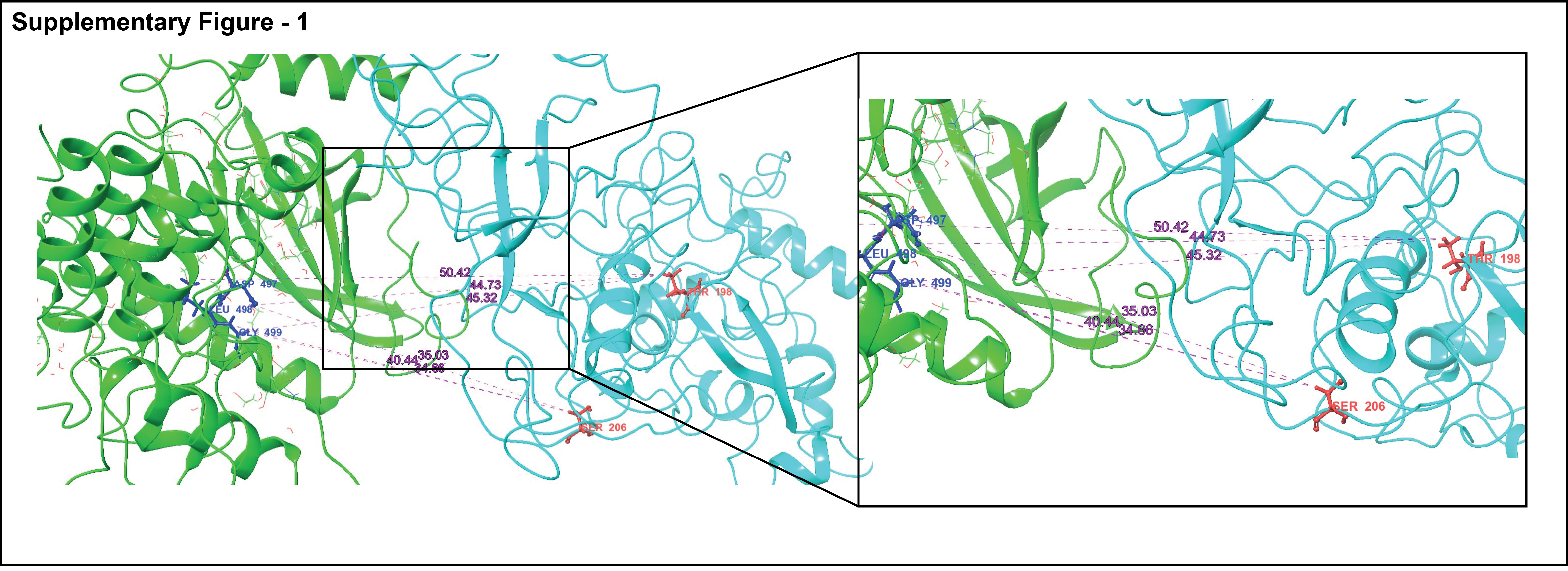

Supplement: Supplementary Figure 1 — Docking results of SRPK1 with experimentally validated phosphorylation sites on the N viral protein at T198 and S206. [file Image1.tif]
